# Experiments and Observations Relating to Various Branches of Natural Philosophy, with a Continuation of Those on Air. The Second Volume

**Published:** 1781-07

**Authors:** 


### THE LONDON MEDICAL JOURNAL, For JULY 1781.

### SECTION I.

### BOOKS.

I. *Experiments and observations relating to various branches of natural philosophy, with a continuation of those on air. The second volume. By* Joseph Priestley, LL. D. F. R. S. *honorary member of the Academy of Sciences at Petersburgh, and of the Royal Academy of Medicine at Paris.* 8vo. Johnson, London, 1781, 408 pages, 6s.

IN the first section of this volume Dr. Priestley has prosecuted the capital discovery which he formerly published of the purification of air by vegetables when exposed to the influence of the sun's light. Mr. Scheele in his treatise on Air and Fire had endeavoured to shew that air is *injured* by vegetation, and that the empyreal or dephlogisticated portion of it is converted thereby into aërial acid. This important ſubject is now cleared up by our illuſtrious author's new experiments.

" In my laſt publication (ſays he) I obſerved

" that the *willow plant* grew very well both in

" inflammable, and in common air, and that it

" abſorbed a conſiderable proportion of both

" the kinds, as well as of nitrous air. In this

" there could not poſſibly be any miſtake, unleſs

" we ſuppoſe the water to have abſorbed the air,

" which it has never been known to do in any

" ſimilar circumſtance. However when I re-

" ſumed the experiments on the growth of this

" plant in the courſe of the laſt ſummer, I had

" no inſtance of the abſorption of common air;

" but I had repeated, and very extraordinary

" ones of the abſorption of inflammable air by

" it; and the plant flouriſhed ſo remarkably in

" it that it may be ſaid to feed upon it with great

" avidity. This proceſs terminates in the change

" of what remains of the inflammable air into

" a new ſpecies of air as good as common air,

" or even better: ſo that it muſt be the inflam-

" mable principle in the air that the plant takes,

" converting it, no doubt, into its proper nou-

" riſhment.

" Some

" Some other plants alſo, as *comfrey*, and *duck-*
" *weed*, I obſerved to thrive very well in in-
" flammable air, and to produce a ſimilar effect
" upon it, though *mint* does not thrive ſo well in
" this as in common air; and I have generally
" found that, in time, this plant is killed by it.

" It may deſerve to be mentioned that the

" willow plant grows beſt in marſhy places which

" abound with inflammable air. The plants

" which I made uſe of grew in the bottom of a

" field, in and near a piece of water, into which,

" if I only thruſt a ſtick a prodigious quantity

" of inflammable air ruſhed out, ſo that, without

" changing my place, I could at any time col-

" lect a large receiver full of it. And bubbles

" of air were very frequently riſing ſpontaneouſly

" from the mud at the bottom.

" It may therefore be a proviſion in nature

" that this noxious kind of air ſhould be fitted

" to the nouriſhment of ſuch plants as grow beſt

" in thoſe places in which it abounds, as well as

" that plants in general ſhould purify the com-

" mon atmoſphere."

The author then proceeds to relate a variety of experiments which confirm the above doctrine, as well as the fact which he had formerly diſ-

covered, that dephlogisticated air is universally hurtful to vegetables.

The second section commences with some observations on the nature of the *green matter* which adheres to the sides of vessels filled with water, when exposed to the sun's light; and which the author had discoursed of in a former volume, as the cause of the purification of the air contained in the water, when assisted by the influence of light. He is now satisfied by experiments that it is a *vegetable* matter: and Mr. Bewly has observed the regular form of it by a microscope. The principal reason which made our author question its being a plant, was, its being produced, as he then thought, in close-stopt vials. But this having been done only with a common cork, the seeds of the plant, which must float insensibly in the air, might have insinuated themselves through some unperceived fracture in it; or they might have been contained in the water previous to its being put into the vial. Both Mr. Bewly and himself found, in the course of the last summer, that when *distilled* water was exposed to the sun in vials filled in part with quicksilver, and in part with distilled water, and inverted in basons of quicksilver, none of this green matter was ever produced; the seeds of the plant not having having been able to penetrate through the mercury to reach the water incumbent upon it, though in ſeveral caſes they inſinuate themſelves in a manner that is truly wonderful.

He next relates experiments which further demonſtrate that the purification of air in theſe caſes depends jointly on the ſun's *light*, and on the *green (vegetable) matter* on the bottom and ſides of the veſſel; and that neither of theſe can effect it without the other.

Dr. Ingenhouſz is of opinion that the dephlogiſticated air is *generated* or *produced* by the plant and the light. But Dr. Prieſtley proves, by a variety of experiments, that theſe do no more than *purify the air already contained in the water and in the atmoſphere.* He concludes this ſection with obſerving, that "air combined with water is "liable to be phlogiſticated by reſpiration, and "to be dephlogiſticated by vegetation, as much "as air in an elaſtic ſtate out of water. For fiſhes "foul the air contained in the water in which they "are confined, and water plants now appear "to purify it. This is, no doubt, one of the "great uſes of weeds, and other aquatic plants, "with which freſh water lakes, and even ſeas "abound, as well as their ſerving for food to a "great number of fiſhes."—He further obſerves,

ſerves, " that water iſſuing from the earth con-

" tains air of an impure kind; that in its paſ-

" ſage the graſs extracts from it its phlogiſton,

" ſo that it then becomes replete with dephlo-

" giſticated air, and unfit for further nutriment

" to vegetables."

In the third ſection the author relates ſome further obſervations on the *green matter*, which he now conſiders as a plant. The ſeeds of it float inviſibly in the air, inſinuate themſelves through the ſmalleſt apertures, yield plants in all ſeaſons of the year, wherever they meet with water; and are capable of producing air in the ſpace of a few days. The richeſt pabulum for it are the putreſcent parts of vegetable and animal ſubſtances. If however the quantity of putrefactive matter in the water be too great it will phlogiſticate the air as faſt as the plants produce it.

Rain water, and diſtilled water, yield the green matter, and conſequently produce air, later than pump water. The latter containing more air to operate upon, and in a leſs pure ſtate. Water impregnated with *fixed air* did not begin to yield the green matter, and pure air, till after the fixed air might be ſuppoſed to have made its eſcape.

The

The fourth and fifth sections treat of the production of the green matter and pure air, by means of various vegetable and animal substances in water. The most remarkable circumstance attending these experiments was that some substances, concerning which the author could have had no such expectation *a priori*, instead of admitting the growth of this plant when they began to putrefy and dissolve (which was the case with most vegetable and animal substances) yielded from themselves a very great quantity of inflammable air; and it made no difference whether they were placed in the sun or in the shade. Whereas other substances, which, if they had been confined by quicksilver, would have yielded, by putrefaction, inflammable air also, together with a portion of fixed air, only supplied that proper pabulum for this green matter, and the whole produce was pure dephlogisticated air; the phlogiston, which in other circumstances would have been converted into inflammable air, now going to the nourishment of this plant, which by the influence of the light yields such pure air. The author then relates a variety of experiments from which these observations were deduced; and concludes the subject with the following judicious reflections:

" It is impoffible not to obferve from thefe experiments the admirable provifion there is in nature to prevent or leffen the fatal effects of putrefaction, efpecially in hot countries, where the rays of the fun are moft direct, and the heat moft intenfe. For whereas animal and vegetable fubftances by fimply putrefying, would neceffarily taint great maffes of air, and render it wholly unfit for refpiration, the fame fubftances putrefying in water fupply a moft abundant pabulum for this wonderful vegetable fubftance, the feeds of which appear to be in all places difperfed invifibly through the atmofphere, and capable, at all feafons of the year, of taking root, and immediately propagating themfelves to the greateft extent. By this means, inftead of the air being corrupted, a vaft addition of the pureft air is continually thrown into it.

By this means alfo, ftagnating waters are rendered much lefs offenfive and unwholefome than they would otherwife be. That froth which alfo we fee on the furface of fuch waters, and which is apt to create difguft, generally confifts of the pureft dephlogifticated air, fupplied by aquatic plants, which always grow in the greateft abundance, and flourifh moft in water that abounds with putrid matter. When the fun fhines, thefe plants plants may alſo be ſeen to emit great quantities of pure air.

Even when animal and vegetable ſubſtances putrefy in *air*, as they have ſome moiſture in them, various other plants in the form of *mold*, &c. find a proper nutriment in them; and by converting a conſiderable part of the phlogiſtic effluvium into their own nutriment, arreſt it in its progreſs to corrupt the ſurrounding atmoſphere. So wonderfully is every part of the ſyſtem of nature formed, that good never fails to ariſe out of all the evils to which, in conſequence of general laws moſt beneficial to the whole, it is neceſſarily ſubject. It is hardly poſſible for a perſon of a ſpeculative turn not to perceive, and admire, this moſt wonderful and excellent proviſion."

[ *To be continued.* ]

